# Analysis of Amino Acid, Vitamin, and Mineral Content in Chinese Gallnut (*Rhus chinensis* Mill.) Honey from Guizhou Province

**DOI:** 10.3390/foods15111943

**Published:** 2026-06-01

**Authors:** Tian Zhao, Changshi Ren, Yinchen Wang, Mengqing Deng, Rongqing Ren, Hua Wang, Yan Liao, Xu Yang, Liming Wu, Xiaofeng Xue, Xiaoming Fang, Yu Fang, Kai Wang

**Affiliations:** 1 Guizhou Institute of Animal Husbandry and Veterinary Science, Guiyang 550002, China; zhaotianyeah@163.com (T.Z.); xmsdmq@163.com (M.D.); mrren2023@163.com (R.R.); wanghua7277@126.com (H.W.); m13981797280@163.com (Y.L.); zixiyang147@163.com (X.Y.); 2 Changshun County Bureau of Agriculture and Rural Affairs, Qiannan Autonomous Prefecture 550700, China; changshia2022@163.com; 3 Apicultural Research Institute, Chinese Academy of Agricultural Sciences, Beijing 100093, China; apiswu@126.com (L.W.); xue_xiaofeng@126.com (X.X.); fangxiaoming@caas.cn (X.F.); fangyu@caas.cn (Y.F.)

**Keywords:** free amino acid, vitamin, mineral, gallnut, Guizhou province, climate

## Abstract

This paper aims to analyze the differences in the contents of free amino acids, minerals, and vitamins in major autumn honey sources—hypothesized to be predominantly derived from Chinese gallnut based on field observations—within Guizhou Province, as well as the correlations between these substances and climatic factors and altitude. We selected honey samples from 10 regions in Guizhou Province and measured their free amino acid, vitamin, and mineral contents. The Kruskal–Wallis test was used to compare differences among regions, and Pearson correlation analysis was used to evaluate the relationships between bioactive compound levels and environmental factors. The total amino acid content ranged from 507.01 ± 3.98 μg/g (Bijiang) to 3056.94 ± 32.88 μg/g (Xifeng); the total vitamin content ranged from 21.94 ± 0.39 μg/g (Xifeng) to 403.67 ± 2.49 μg/g (Zheng’an). The total mineral content ranged from 42.44 ± 0.37 μg/g (Xifeng) to 98.09 ± 1.51 μg/g (Bijiang). Vitamin K_1_, vitamin B_1_, vitamin D_3_, and selenium were not detected in any of the honey samples. Mean precipitation showed a significant positive correlation with total free amino acid content and total mineral content (*p* < 0.05); the Normalized Difference Vegetation Index (NDVI) was positively correlated with total vitamin content (*p* < 0.05); and temperature was positively correlated with total mineral content (*p* < 0.05). The study suggests that multiple factors, including climatic and geographical factors, collectively influence the bioactive substance profiles of honey, providing insights into the origin authentication and quality trends of this regional product.

## 1. Introduction

Honey is a natural sweet substance produced by honeybees by collecting plant nectar, secretions, or honeydew, mixing them with their own secretions, and allowing the mixture to ripen in the honeycomb fully. Research has shown that honey exhibits significant antioxidant, antibacterial, anti-inflammatory, antimicrobial, antidiabetic, and wound-healing and sunburn-healing activities, and also helps assess the presence of environmental pollutants [1,2,3,4,5,6]. Increasingly, honey is recognized not only as a nutritional product of economic value but also as a valuable matrix for monitoring environmental quality and food safety [7]. In recent years, honey has gained widespread attention and research interest due to its unique flavor, high nutritional value, and health benefits. Chemically, honey consists primarily of sugars and water, along with small but important amounts of amino acids, minerals, vitamins, and other substances. The composition and proportions of these compounds determine the differences in color, taste, and nutritional value of each type of honey [5,8,9]. The composition of these substances is influenced by factors such as environment, geographical conditions, plant species, and processing and storage methods [10], which contribute to the diversity of honey varieties and their market performance.

Guizhou Province is located in southwestern China and features a shell-shaped topography: high and relatively arid in the northwest, and lower and relatively humid in the surrounding areas. The terrain is characterized by rolling mountains and significant elevation differences, making it a typical karst landform region. The province has a predominantly subtropical monsoon climate, with warm winters and cool summers, and a rainy season concentrated in July when the forests are lush, exhibiting a pattern of concurrent rain and heat. The gallnut tree, an autumn-blooming plant widely distributed across low, medium, and high altitudes in Guizhou, serves as a good local autumn nectar source. Chinese gallnut is a commonly used traditional Chinese medicinal material recorded in the *Pharmacopoeia of the People’s Republic of China*, with a long history of production and application. As early as over 2000 years ago, it was documented in the *Classic of Mountains and Seas*, and its uses were recorded in the *Illustrated Classic of Materia Medica* and *Kaibao Materia Medica* of the Song Dynasty, the *Compendium of Materia Medica* of the Ming Dynasty, and the *New Compilation of Materia Medica* of the Qing Dynasty [11]. The chemical components of Chinese gallnut honey include tannins, gallic acid, oleic acid, linoleic acid, lauric acid, linolenic acid, and trace elements such as iron, copper, zinc, and calcium [12]. Honey produced by bees collecting nectar from this plant is amber in color, slightly bitter in taste, and possesses a strong gallnut aroma. This honey is an important varietal in Guizhou Province, harvested in large quantities during autumn, and highly favored by consumers.

Previous studies on amino acids, vitamins, and minerals in honey have included qualitative and quantitative analyses conducted by several authors. For instance, Yang et al. [10] found that the types of amino acids in Chinese honey from the Chongqing region vary among different honey varieties. While no significant differences were observed in total free amino acids, essential amino acids, or non-essential amino acids, a significant difference was noted in proline content. Analysis of 13 honey samples revealed that amino acid content varies across honey varieties, with total amino acid content ranging from 0.107% to 1.126%. Among the varieties, fennel honey exhibited the highest total amino acid content, followed by local wild honey, then mountain flower honey, chrysanthemum honey, and wolftooth honey, while rapeseed honey and linden honey had relatively lower contents. Fennel honey showed higher levels of Asp, Pro, Phe, Thr, Ser, Glu, Gly, Ala, Cys, Val, Ile, Leu, and Lys. Local wild honey exhibited higher levels of His and Tyr, while wolftooth honey contained a higher level of Met [13]. The sum of 20 amino acids in five types of specialty honey—*Macadamia integrifolia* honey, *Coffea* honey, *Leucosceptrum canum* Smith honey, *Vicia villosa* Roth honey, and *Hevea brasiliensis* honey—ranges from 312.54 to 1245.51 mg/kg. The order of total amino acid content is as follows: *Coffea* honey > *L. canum* Smith honey > *V. villosa* Roth honey > *H. brasiliensis* honey > *M. integrifolia* honey. The composition and content of amino acids vary significantly among different honey varieties. Notably, the tyrosine content in *Coffea* honey is more than ten times that found in other types of honey [14]. Fan et al. [15] examined Chinese gallnut honey from 14 regions and found that the total free amino acid content ranged from 0.011 g/100 g to 0.034 g/100 g. Among these samples, the highest total free amino acid content was observed in Baiyun, Guizhou, while the lowest was found in Mayang, Hunan. Proline content also exhibited regional variation. In addition, honey samples collected from the southwestern and northeastern regions of Shennongjia showed considerable differences in both total amino acid and proline content. Shi et al. [16,17] determined six vitamins and four mineral elements in Xinjiang black bee honey from Nileke. Notably, fat-soluble vitamins were not detected in samples from certain regions. Escuredo et al. [18] determined mineral elements including iron, copper, magnesium, zinc, and phosphorus in chestnut honey from the northwest region of Spain. The mineral content of Chinese gallnut honey from eight counties in Guizhou Province showed regional differences, with lower mineral content observed in the flowers of Chinese gallnut honey from Suiyang, Wangmo, Dushan, and Huangping [15,19]. Blueberry honey has a low total mineral content but a rich diversity of mineral species, with copper being present only in blueberry honey. The total mineral content of wild rose honey is significantly higher than that of blueberry honey, and it is characterized by high calcium, high potassium, and low sodium levels [20]. These studies, on the one hand, have not addressed the unique topographical and climatic conditions within Guizhou Province, particularly given that Chinese gallnut honey is a dominant autumn nectar source in the region. On the other hand, when analyzing active components, these studies have incorporated an insufficient range of influencing factors, failing to account for variables such as temperature, precipitation, vegetation, and altitude. Therefore, a systematic analysis of the amino acid, vitamin, and mineral contents of honey that integrates meteorological and geographical factors is not only crucial for elucidating its characteristics but also holds significant economic value for the development of its functional components.

Based on these research gaps, the main objectives of this study are: (1) to systematically quantify and compare the levels of free amino acids, vitamins, and minerals in Chinese gallnut honey from 10 distinct geographical regions within Guizhou Province; (2) to investigate the correlations between the bioactive compound profiles (total and individual compounds) and key environmental and geographical factors, namely mean precipitation and temperature, NDVI, and elevation; and (3) to provide a scientific basis for the quality standardization, geographical origin authentication, and sustainable development of this unique honey product.

In conclusion, this study attempts to analyze Chinese gallnut honey from different regions of Guizhou Province by integrating collected climatic data, altitude, and vegetation. It aims to uncover the underlying mechanisms governing the quality formation of Guizhou Chinese gallnut honey. This will, in turn, provide a theoretical foundation and data support for the standardization of quality, the protection of geographical indications, and the sustainable development of the industry.

## 2. Materials and Methods

### 2.1. Honey Source and Storage

Based on breeding observations and feedback from local beekeepers, we selected areas with abundant Chinese gallnut trees in Guizhou Province. We hypothesized that honey collected from hives in these areas during this specific period would be predominantly unifloral Chinese gallnut. These 10 regions include Longli County (LL), Dushan County (DS), Taijiang County (TJ), Duyun City (DY), Xifeng County (XF), Fenggang County (FG), Bijiang District (BJ), Majiang County (MJ), Zheng’an County (ZA), and Kaiyang County (KY). The altitudes of the collection sites, in descending order, are as follows: KY(1350.4 m), LL (1298.3 m), FG (1104.5 m), ZA (1027.6 m), DS (998.4 m), MJ (916.7 m), DY (884.9 m), TJ (878.8 m), XF (621.1 m), and BJ (285.0 m). These samples were collected during the first two weeks of October 2023. At that time, there were very few flowering plants in these areas, with practically only the gallnut tree in bloom. In each of the 10 regions, we selected 3 independent apiaries, each about 3 km apart, for a total of 30 apiaries. Within each apiary, mature, capped honey was extracted from at least 3 different beehives and pooled to obtain one representative composite sample (≥1 kg) per apiary. The apiaries are located away from residential areas, situated either within forested mountains or at the border between forests and abandoned farmland. These apiaries use modern movable-frame beekeeping. After collecting, the honey samples were stored in a car refrigerator until being transferred to a laboratory freezer (−20 °C). All these honey samples were capped mature honey with a sugar content exceeding 41 °Bé. Although the Chinese gallnut was overwhelmingly dominant during this period, it should be noted that the unifloral nature of the honey samples was not analytically verified by melissopalynology or DNA metabarcoding, which constitutes a limitation of the current study.

### 2.2. Determination of Free Amino Acids

The content of free amino acids (Aspartic acid, Glutamic acid, Asparagine, Serine, Glutamine, Glycine, Histidine, Arginine, Threonine, Alanine, Proline, Tyrosine, Valine, Methionine, Cystine, Isoleucine, Leucine, Phenylalanine, Lysine) was determined according to the latest Chinese standard (SN/T 5223—2019). Namely, 3.0 g of each honey sample was accurately weighed, dissolved by vortex mixing with an appropriate volume of ultrapure water, and then diluted to a final volume of 25 mL with ultrapure water. The solution was filtered through a 0.22 μm membrane filter prior to injection into a High-Performance Liquid Chromatography (HPLC) system. Bioactive compound analysis was performed by Biomarker Cloud Technology Co., Ltd. (Beijing, China). Detailed testing and calculations can be found in Supplementary Table S1.

### 2.3. Determination of Vitamin Content

Vitamin content was determined using a Thermo Scientific Dionex UltiMate 3000 High-Performance Liquid Chromatography (HPLC) system equipped with a Syncronis C18 column (Dimensions: 250 mm × 4.6 mm, particle size: 5 μm). The contents of vitamins (vitamin A, β-carotene, vitamin B_1_, vitamin B_2_, vitamin B_6_, vitamin B_12_, pantothenic acid, niacin, folic acid, total L(+)-ascorbic acid, vitamin D_3_, vitamin E, α-tocopherol, γ-tocopherol, δ-tocopherol, biotin, vitamin K) in each honey type were strictly determined according to the relevant Chinese National Standards listed in Supplementary Table S1. Method validation was performed for linearity, precision, recovery, and sensitivity. Calibration curves for all target vitamins showed good linearity (R^2^ > 0.995) within their respective calibration ranges. The limits of detection (LOD) and quantification (LOQ) were determined based on signal-to-noise ratios of 3 and 10, respectively. Method precision, expressed as relative standard deviation (RSD), was below 10% for all analytes. Recovery rates, assessed through spiked blank matrix experiments, fell within the acceptable range of 85% to 110%. A substance was reported as ‘not detected’ (ND) when its concentration fell below the LOD.

### 2.4. Determination of Mineral Content

Mineral content was determined using Inductively Coupled Plasma Optical Emission Spectrometry (ICP-OES, Thermo Scientific iCAP 7400) (Waltham, MA, USA). The contents of minerals (calcium, iron, magnesium, zinc, copper, potassium, sodium, manganese, phosphorus, selenium) in each honey type were strictly determined according to the relevant Chinese National Standards listed in Supplementary Table S1. The method was validated with multi-element reference standards. Calibration curves had R^2^ > 0.999. LODs and LOQs for each mineral were established based on the standard deviation of the blank and the slope of the calibration curve. Precision (RSD) was below 5% for all elements, and spike recovery rates were between 90% and 110%. Selenium levels were below the LOD in all samples and thus reported as ND.

### 2.5. Temperature, Precipitation, NDVI, and Elevation

Although the overall temperature and precipitation cannot fully reflect the actual conditions of the collection sites, considering that Chinese gallnut concentrates its flowering and nectar secretion during this period, We selected the mean precipitation, mean temperature, and the maximum Normalized Difference Vegetation Index (NDVI)—a satellite-derived metric of vegetation density and health—for the pre-honey-harvest period of 2023 to reflect the air temperature, humidity conditions, and vegetation status of the collection sites. These pre-honey-harvest data from 2023 were selected because they influence the early growth and flowering of the Chinese sumac tree. The mean temperature and precipitation data for the pre-honey-harvest period of October 2023 were obtained from the National Oceanic and Atmospheric Administration (NOAA, https://www.noaa.gov/) (accessed on 16 March 2024). The NDVI values represent the 2023 annual maximum. The elevation data were measured using a mobile GPS application. In the following description, we will collectively refer to these three meteorological values and elevation as “environmental factors”. Detailed values are presented in Table 1.

### 2.6. Statistical Analysis

The test data were organized using Microsoft Excel. For all statistical comparisons, honey samples were grouped by region, and the regional mean was used as the unit of analysis. This acknowledges that the environmental variables (Table 1) are region-specific, not apiary-specific. Data were analyzed using the Kruskal–Wallis test, and *p*-values were corrected using the Bonferroni method. Statistical significance was set at 95% or 99%. Pearson correlation analysis of bioactive compounds and environmental factors was performed in R 4.5.2, with the psych package used for multiple-comparison correction (Bonferroni method) and the corrplot package for inter-group correlation analysis. A multiple linear regression (MLR) model was built to explore the combined relationship between environmental factors and proline content. Prior to correlation analysis, the experimental results were normalized. Results were visualized using the ggplot2 package. All results are presented as mean ± standard deviation.

## 3. Results and Discussion

### 3.1. Free Amino Acid Content and Its Relationship with Environmental Factors

The free amino acids content in samples ranged from 507.01 ± 3.98 μg/g (BJ) to 3056.94 ± 32.88 μg/g (XF). The three amino acids with the highest contents were as follows: Pro levels ranged from 113.97 ± 3.6 μg/g (DS) to 696.19 ± 5.45 μg/g (BJ); Phe levels ranged from 62.07 ± 2.07 μg/g (FG) to 1680.39 ± 14.93 μg/g (BJ); Met levels ranged from 16.14 ± 3.04 μg/g (KY) to 382.95 ± 21.22 μg/g (ZA) (Supplementary Table S2). The proportion of Pro in all samples ranged from 8.11% to 28.90% (Supplementary Table S3). The Kruskal–Wallis test, followed by Bonferroni-corrected pairwise comparisons, was used to identify significant differences in individual amino acid contents among the 10 regions (Table 2).

Amino acids are a relatively important class of minor components in honey. Several amino acids, including proline, are associated with antioxidant capacity; therefore, some authors consider proline content a key factor in evaluating honey quality [21,22]. In this study, we determined 19 amino acids (ranging from 0.51 to 3.10 mg/g). Four essential and four non-essential amino acids were identified, among which phenylalanine was the most abundant amino acid in most honey samples (mean content: 0.0874 g/100 g). Only in the samples from Fenggang and Xifeng was the Phe content lower than the Pro content. Previous studies have reported that the content of 18 amino acids in honey from honeybees and bumblebees ranged from 0.02 to 44.41 mg/100 g [21]; and Chinese gallnut honey contains 12 amino acids (1.32–2.92 mg/g), with phenylalanine being the most abundant, showing an average content of 0.0836 g/100 g, which is significantly higher than that of proline (0.0291 g/100 g) [19]. These findings are generally consistent with the upper limit of total amino acid content observed in this study. However, the minimum values we detected were lower, indicating that our sample selection was more comprehensive and better reflects the actual geographical variation within Guizhou Province. A recent larger-scale study combining free amino acid profiling, chemometrics, and DNA-based plant identification on 93 Chinese honey samples also demonstrated the power of amino acid fingerprints for floral and geographical discrimination, providing a robust benchmark for our findings [22].

After *p*-value correction, no significant difference in Ala content was found among the 10 groups. Although the overall difference in Arg content was significant, pairwise comparisons revealed no significant differences between groups, possibly due to the small sample size limiting statistical power. For Pro content, the significance between DS and BJ was less than 0.01; between DS and ZA, it was less than 0.05; and between DY and BJ, it was less than 0.05. The results of the intergroup comparisons of amino acid content are presented in Table 2.

**Figure 1:**
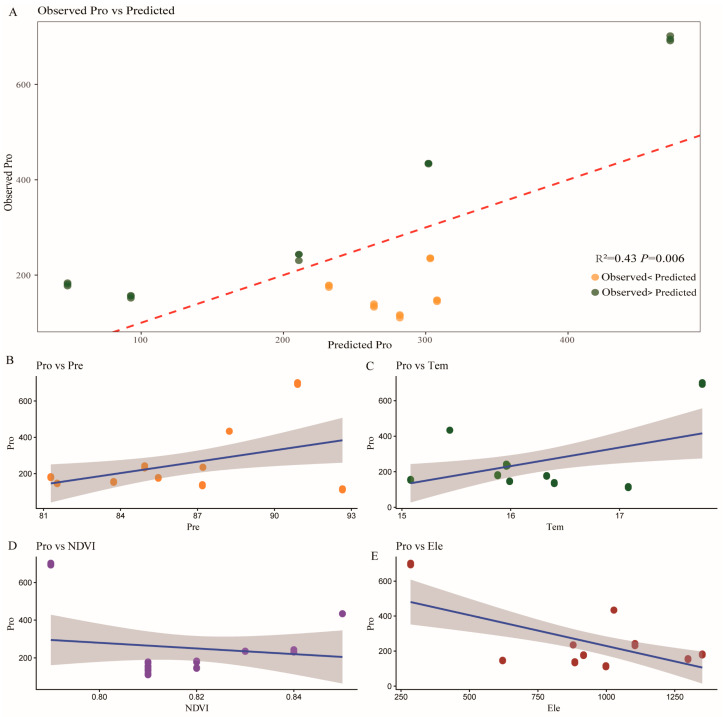
Scatter plot of multiple regression between Pro and climatic factors (**A**) and regression plots between Pro and the other four factors (**B**–**E**). Notes: Pre: annual mean precipitation; Tem: annual mean temperature; Ele: elevation of the collecting site.

A multiple linear regression (MLR) model was constructed to explore the combined relationship between the four environmental factors and Pro content. A scatter plot of the MLR-predicted Pro values against the actual observed Pro values indicated a positive correlation (R^2^ = 0.43, *p* = 0.006; Figure 1), suggesting that these environmental factors may collectively account for a proportion of the variance in Pro content across regions. However, given the limited sample size (n = 10) and the exploratory nature of this analysis, these results should be interpreted with caution, and further validation with larger datasets is warranted. From the regression results of Pro with the other individual climatic factors, the trend of Pro was consistent with that of Pre and Tem, while it showed opposite trends with NDVI and Ele.

**Figure 2:**
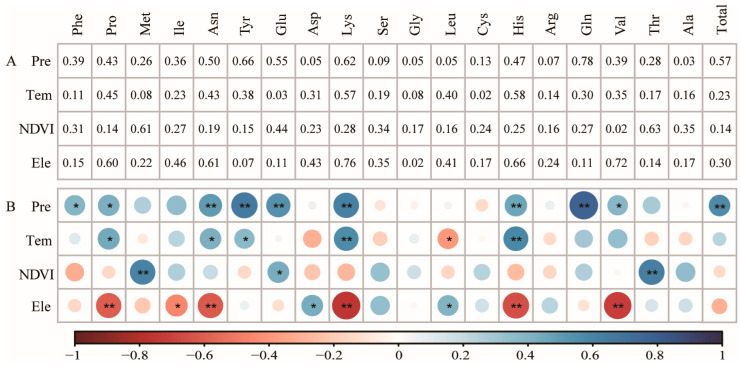
The absolute values of correlation coefficients between amino acids and environmental factors (**A**) and the figure (**B**). Notes: Pre: annual mean precipitation; Tem: annual mean temperature; Ele: elevation of the collecting site. The * and ** represent *p* < 0.05 and *p* < 0.01, respectively.

Pre showed significant positive correlations with the total free amino acids (*p* < 0.05). Specifically, Pre was positively correlated with Phe (r = 0.39), Pro (r = 0.43), Asn (r = 0.50), Tyr (r = 0.66), Glu (r = 0.55), Lys (r = 0.62), His (r = 0.47), Gln (r = 0.78), and Val (r = 0.39). Tem was positively correlated with Pro (r = 0.45), Asn (r = 0.43), Tyr (r = 0.38), Lys (r = 0.57), and His (r = 0.58), while negatively correlated with Leu (r = −0.40). The NDVI showed positive correlations with Met (r = 0.61), Glu (r = 0.44), and Thr (r = 0.63). The Ele was negatively correlated with Pro (r = −0.60), Ile (r = −0.46), Asn (r = −0.61), Lys (r = −0.76), His (r = −0.66), and Val (r = −0.72), while positively correlated with Asp (r = 0.43) and Leu (r = 0.41) (Figure 2).

Honey reflects factors such as vegetation, climate, and altitude. Climatic effects, as an integrated environmental factor, influence the variations in all the aforementioned components. Warm, dry climates promote the accumulation of secondary metabolites in nectar-producing plants, thereby increasing phenolic acid and flavonoid levels and enhancing antioxidant activity [23]. Conversely, rainy or low-temperature climates may lead to nectar dilution, reducing phenolic compound contents [24]. The positive correlations observed in this study between precipitation and several amino acids, notably proline, suggest that adequate water availability during the flowering period of Chinese gallnut may play a role in nitrogen metabolism and amino acid synthesis in the plant or in the regulation of nectar secretion, potentially increasing their concentration in nectar. The generally negative correlations of many amino acids with elevation further indicate that the harsher growing conditions (lower temperatures, shorter growing seasons) at higher altitudes within Guizhou may reduce the plant’s capacity for synthesizing these nitrogenous compounds, a pattern that has been observed in other plant systems. According to previous reports, honey stored under light conditions exhibits a faster rate of HMF accumulation and a more rapid decline in antioxidant activity compared to samples stored in darkness [23]. Low temperatures in the climate affect enzymes such as amylase, D-glucose-1-oxidase, and catalase. [25]. Excessive retention time of honey within the hive can also significantly degrade its quality [26,27]. Even the container used for honey storage affects its physicochemical properties [28]. Human activities, such as industrial operations, traffic emissions, and coal combustion, release toxic heavy metals, including lead, cadmium, mercury, and arsenic, into the atmosphere. These particulates can enter soil and water bodies through dry and wet deposition, be absorbed by nectar plants and translocated to nectar and pollen, or directly adhere to plant surfaces and be brought into the hive by foraging bees [29,30,31]. Notably, although honeybees possess a certain biofiltration capacity that can partially block the transfer of heavy metals to honey, long-term exposure may still lead to pollutant accumulation in honey [32,33,34,35]. This physiological stress may alter bee foraging behavior, subsequently affecting honey yield and composition. Furthermore, nectar plants under pollution stress may exhibit stress responses, altering the sugars, phenolic compounds, and enzyme activities in nectar, thereby affecting honey’s antioxidant capacity and flavor [36]. Banned pollutants such as organochlorine pesticides, polychlorinated biphenyls (PCBs), and polybrominated diphenyl ethers (PBDEs) have also been detected in honeybees, bee bread, beeswax, and honey, indicating comprehensive contamination of bee products by agricultural and industrial activities [37].

### 3.2. Vitamins and Minerals Content and Their Relationship with Environmental Factors

Overall, the total vitamin content in the ten honey samples ranged from 21.94 ± 0.39 μg/g (XF) to 403.67 ± 2.49 μg/g (ZA). Ascorbic acid content ranged from 6.22 ± 0.42 μg/g (XF) to 143.41 ± 0.87 μg/g (BJ). Niacin content ranged from 0.64 ± 0.02 μg/g (BJ) to 2.66 ± 0.09 μg/g (MJ). Folic acid content ranged from 0.36 ± 0.03 μg/g (FG) to 56.05 ± 1.10 μg/g (DS). Vitamin B_6_ content ranged from 1.95 ± 0.05 μg/g (DS) to 130.49 ± 0.81 μg/g (FG). γ-Tocopherol content ranged from 0.11 ± 0.01 μg/g (FG) to 1.42 ± 0.04 μg/g (TJ). Vitamin E content ranged from 0.11 ± 0.01 μg/g (FG) to 3.00 ± 0.08 μg/g (TJ). Among the 17 vitamins analyzed, ascorbic acid, niacin, folic acid, vitamin B_6_, γ-tocopherol, and vitamin E were detected in all samples. Some vitamins were not detected (Supplementary Table S4). We selected these six vitamins, all commonly found in honey, for comparison. Significant differences in the contents of the six commonly detected vitamins among regions were determined using the Kruskal–Wallis test, with results of pairwise comparisons detailed in Table 3.

The total vitamin content varied considerably across honey samples from 10 regions, with six vitamins present in all samples. The correlation coefficients between total vitamin content and Pre, Tem, NDVI, and Ele are 0.33 (*p* > 0.05), −0.11 (*p* > 0.05), 0.58 (*p* < 0.05), and 0.05 (*p* > 0.05), respectively. Previous studies have indicated that the composition and concentration of vitamins in honey are determined by the chemical composition of nectar and pollen from nectar-producing plants and are significantly influenced by soil, climate, and weather conditions [38]. A study on honeys from different altitudes and floral sources found that the concentration of vitamin C in honey is positively correlated with the content of dicarbonyl molecules but negatively correlated with hydrogen peroxide content, suggesting that both bee metabolic processes and environmental factors jointly shape the vitamin profile of honey [39]. Considering that the flowering of Chinese gallnut is related to altitude, and that vitamin content was higher in low- and mid-altitude regions in this study, we speculate that the total vitamin content is influenced by the combined effects of precipitation and temperature, driven by altitude.

The total mineral content ranged from 42.44 ± 0.37 μg/g (XF) to 98.09 ± 1.51 μg/g (BJ) (Supplementary Table S5). Significant differences in each mineral among the groups were determined using the Kruskal–Wallis test, with results of pairwise comparisons detailed in Table 4. Mg content ranged from 9.84 ± 0.12 μg/g (LL) to 35.95 ± 0.22 μg/g (XF). Mn content ranged from 0.17 ± 0.01 μg/g (BJ) to 1.90 ± 0.02 μg/g (FG). Fe content ranged from 3.53 ± 0.10 μg/g (KY) to 6.81 ± 0.03 μg/g (TJ). Cu content ranged from 0.05 ± 0.01 μg/g (DY) to 0.16 ± 0.01 μg/g (TJ). Zn content ranged from 1.19 ± 0.02 μg/g (KY) to 2.05 ± 0.06 μg/g (BJ). Na content ranged from 12.43 ± 0.02 μg/g (DS) to 20.98 ± 0.10 μg/g (MJ). Among all honey samples, K content was the highest, ranging from 141.17 ± 3.99 μg/g (XF) to 540.33 ± 7.20 μg/g (BJ). Ca content ranged from 44.39 ± 0.13 μg/g (DS) to 103.28 ± 0.46 μg/g (FG). P content ranged from 54.20 ± 1.14 μg/g (KY) to 231.98 ± 9.05 μg/g (FG). Selenium was not detected in any of the samples. Pairwise comparisons of each mineral content are presented in Table 4.

Studies on mineral content in honey have shown that mineral levels vary across regions, with differences evident in both total mineral content and the types of minerals present [20]. For example, four mineral elements were determined in Xinjiang black bee honey [16], which differs from chestnut honey [18]. Even within the same honey type, regional differences in mineral content have been observed [15,19]. In this study, the total mineral content was similar to that reported by other authors; however, some differences were noted in the types of minerals detected. We hypothesize that these differences are primarily related to the soil mineral composition of the respective regions.

**Figure 3:**
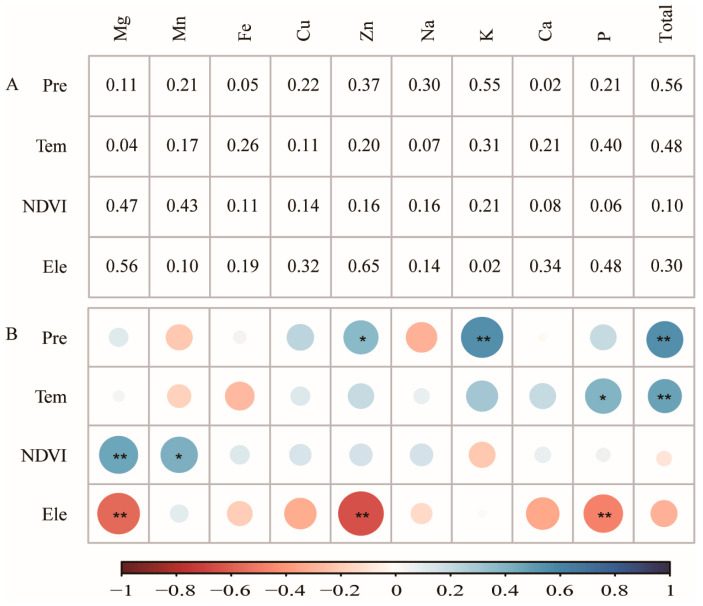
The absolute values of correlation coefficients between minerals and environmental factors (**A**) and the figure (**B**). Notes: Pre: annual mean precipitation; Tem: annual mean temperature; Ele: elevation of the collecting site. The * and ** represent *p* < 0.05 and *p* < 0.01, respectively.

The total mineral content showed significant correlations with Pre and Tem (r = 0.56 and 0.48, respectively, *p* < 0.05). The correlation analysis showed that Pre was significantly positively correlated with Zn and K contents (r = 0.37 and 0.55, respectively). Tem was significantly positively correlated with P content (r = 0.40). NDVI was significantly positively correlated with Mg and Mn contents (r = 0.47 and 0.43, respectively). Ele was significantly negatively correlated with Mg, Zn, and P contents (r = −0.56, −0.65, and −0.48, respectively) (Figure 3).

Seasonal changes have been demonstrated to be a key factor affecting the physicochemical properties and antioxidant capacity of stingless bee honey [40]. Geographical origin, which represents different climatic conditions, not only affects basic quality parameters such as moisture content and sugar ratio but also significantly influences non-quality parameters, including pH and enzyme activity [40]. In this study, mean precipitation showed a significant association with both total free amino acid content and total mineral content; NDVI was positively correlated with total vitamin content; and temperature was positively correlated with total mineral content. However, overall, the correlations of precipitation, temperature, and NDVI with total amino acid, mineral, and vitamin contents were not particularly strong (ranging from 0.48 to 0.58). Furthermore, altitude did not exhibit a significant correlation with these total contents but did show significant correlations with certain specific categories (e.g., lysine with elevation).

### 3.3. Other Factors Potentially Influencing Bioactive Compound Profiles

Several unmeasured environmental and management factors likely contribute to the unexplained variance in our data. For instance, soil mineral composition and geochemical background can directly influence the mineral content of nectar and, consequently, honey [29,30,31]. Although the apiaries in this study were situated in forested or forest-edge habitats at a distance from urban centers and intensive farming, they were still within a broader regional landscape influenced by human activities. Potential exposure to airborne pollutants cannot be entirely excluded. Furthermore, post-harvest handling, such as storage temperature and light exposure, can affect labile vitamins like ascorbic acid and degrade antioxidant compounds [25,26,27,28]. These factors should be systematically quantified in future studies to build more robust explanatory models.

In addition, our identification of the honey as gallnut honey relied on the dominant flower species in the field and beekeeper experience, rather than melissopalynological or DNA-based confirmation. As highlighted by recent methodological advances, combining microscopic pollen analysis with DNA metabarcoding provides the most reliable approach for verifying honey’s floral origin [41]. This is a critical area for improvement in follow-up research, as the precision of correlations between specific nectar sources and environmental drivers is entirely dependent on accurate botanical assignment.

Furthermore, the effects of bee foraging behavior and physiology should not be overlooked. When collecting nectar, workers do not engage in passive gathering; instead, they actively perform energetic accounting. During this process, pre-processing during flight and the addition of invertase after returning to the hive catalyze the formation of honey. The gut microbiome of bees may also play an auxiliary role in shaping the final quality of honey [42]. Although the high osmotic pressure and low pH of honey itself inhibit the growth of most microorganisms, during the early stages of the transition from nectar to honey, the bee gut symbiotic microbiota may participate in the breakdown of certain complex carbohydrates and the formation of flavor compounds [43]. These biological processes introduce an additional layer of variability between the plant’s nectar composition and the final honey product, further emphasizing the need for a systems-level approach in future studies. According to previous reports, honey stored under light conditions exhibits a faster rate of HMF accumulation and a more rapid decline in antioxidant activity compared to samples stored in darkness [23]. Low temperatures in the climate affect enzymes such as amylase, D-glucose-1-oxidase, and catalase. [25]. Excessive retention time of honey within the hive can also significantly degrade its quality [26,27]. Even the container used for honey storage affects its physicochemical properties [28]. Human activities, such as industrial operations, traffic emissions, and coal combustion, release toxic heavy metals, including lead, cadmium, mercury, and arsenic, into the atmosphere. These particulates can enter soil and water bodies through dry and wet deposition, be absorbed by nectar plants and translocated to nectar and pollen, or directly adhere to plant surfaces and be brought into the hive by foraging bees [29,30,31]. Notably, although honeybees possess a certain biofiltration capacity that can partially block the transfer of heavy metals to honey, long-term exposure may still lead to pollutant accumulation in honey [32,33,34,35]. This physiological stress may alter bee foraging behavior, subsequently affecting honey yield and composition. Furthermore, nectar plants under pollution stress may exhibit stress responses, altering the sugars, phenolic compounds, and enzyme activities in nectar, thereby affecting honey’s antioxidant capacity and flavor [36]. Banned pollutants such as organochlorine pesticides, polychlorinated biphenyls (PCBs), and polybrominated diphenyl ethers (PBDEs) have also been detected in honeybees, bee bread, beeswax, and honey, indicating comprehensive contamination of bee products by agricultural and industrial activities [37].

## 4. Conclusions

This study provides a comprehensive comparative analysis of free amino acid, vitamin, and mineral profiles in Chinese gallnut honey from ten regions across Guizhou Province. Significant regional variations were observed. Phenylalanine, proline, ascorbic acid, and potassium were the predominant components in their respective categories. These profiles were significantly correlated with environmental factors—precipitation was positively associated with total free amino acid and mineral contents, NDVI with total vitamin content, and temperature with total mineral content—while elevation showed negative correlations with specific minerals.

The novelty of this work lies in its integrated approach, simultaneously quantifying multiple bioactive compound classes in a regionally specific honey and systematically linking their composition to climatic and geographical factors. The distinct regional patterns observed provide a scientific foundation for quality standardization, geographical indication protection, and origin authentication of Guizhou gallnut honey. However, the moderate strength of the observed correlations indicates that other unmeasured factors—soil composition, beekeeping practices, and post-harvest handling—also play important roles. Future research should employ melissopalynological and DNA metabarcoding techniques to rigorously confirm monofloral origin, incorporate additional variables into multivariate models, and validate findings through multi-year sampling designs.

## Figures and Tables

**Table 1:** The mean temperature, precipitation, elevation, and maximum NDVI values at the collection sites.

Site	Temperature (°C)	Precipitation (mm)	NDVI	Elevation (m)
BJ	17.76 (290.91 K)	90.90	0.79	285.00
DY	16.40 (289.55 K)	87.19	0.81	884.90
DS	17.08 (290.23 K)	92.65	0.82	998.40
FG	15.96 (289.11 K)	84.94	0.84	1104.50
KY	15.88 (289.03 K)	81.28	0.83	1350.40
LL	15.08 (288.23 K)	83.73	0.85	1298.30
MJ	16.33 (289.48 K)	85.47	0.80	916.70
TJ	15.97 (289.12 K)	87.21	0.82	878.80
XF	15.99 (289.14 K)	81.53	0.86	621.10
ZA	15.44 (288.59 K)	88.24	0.85	1027.60

Notes: Longli County (LL), Dushan County (DS), Taijiang County (TJ), Duyun City (DY), Xifeng County (XF), Fenggang County (FG), Bijiang District (BJ), Majiang County (MJ), Zheng’an County (ZA), and Kaiyang County (KY). NDVI: Normalized Difference Vegetation Index.

**Table 2:** *p*-values for pairwise comparisons of free amino acid content.

Free Amino Acid	*p*-Value for Comparison Between Groups
Phe	FG~BJ < 0.01; BJ~XF < 0.05
Pro	DS~BJ < 0.01; DS~ZA < 0.05; DY~BJ < 0.05
Met	KY~FG < 0.05; KY~ZA < 0.01; DS~ ZA < 0.05
Ile	KY~BJ < 0.05; KY~FG < 0.01; LL~FG < 0.05
Asn	LL~BJ < 0.01; KY~BJ < 0.05
Tyr	XF~TJ < 0.05; XF~DS < 0.01; FG~DS < 0.05
Glu	XF~BJ < 0.05; XF~ZA < 0.01
Asp	DY~DS < 0.05; DY~LL < 0.01
Lys	KY~ZA < 0.05; KY~BJ < 0.01; DY~BJ < 0.05
Ser	DY~ZA < 0.05; DY~KY < 0.01
Gly	LL~ZA < 0.05; LL~KY < 0.01; DS~KY < 0.05
Leu	XF~LL < 0.01; FG~LL < 0.05
Cys	XF~FG < 0.05
His	LL~BJ < 0.05
Gln	LL~ZA < 0.05; LL~DS < 0.01; XF~DS < 0.05
Val	MJ~BJ < 0.05
Thr	DY~ZA < 0.05; MJ~ZA < 0.05

Notes: The symbol “~” connecting two items indicates that the two are compared pairwise. For example, FG~BJ < 0.01 indicates that the *p*-value between the FG and BJ groups is <0.01. The rest can be interpreted accordingly.

**Table 3:** *p*-values for pairwise comparisons of vitamin content.

Vitamins	*p*-Value for Comparison Between Groups
Ascorbic Acid	XF~TJ < 0.05; XF~BJ < 0.01; KY~BJ < 0.05
Niacin	BJ~TJ < 0.05; BJ~MJ < 0.05; ZA~MJ < 0.05
Folic Acid	FG~DS < 0.01; XF~DS < 0.05
Vitamin B_6_	DS~ZA < 0.05; DS~FG < 0.01; KY~FG < 0.05
γ-Tocopherol	FG~TJ < 0.05; DS~TJ < 0.05
Vitamin E	FG~BJ < 0.05; FG~TJ < 0.01; DS~TJ < 0.05

Notes: The symbol “~” connecting two items indicates that the two are compared pairwise. For example, XF~TJ < 0.05 indicates that the *p*-value for the difference between the XF and TJ groups is <0.05. The rest can be interpreted accordingly.

**Table 4:** *p*-values for pairwise comparisons of mineral content.

Minerals	*p*-Value for Comparison Between Groups
Mg	LL~XF < 0.05; KY~XF < 0.05
Mn	BJ~MJ < 0.05; BJ~FG < 0.01; LL~FG < 0.05
Fe	KY~LL < 0.05; KY~TJ < 0.01; DS~TJ < 0.05
Cu	DY~BJ < 0.05; DY~TJ < 0.01
Zn	KY~TJ < 0.05; KY~BJ < 0.05
K	XF~TJ < 0.05; XF~BJ < 0.01; FG~BJ < 0.05
Ca	DS~BJ < 0.05; DS~FG < 0.01; MJ~FG < 0.05
P	KY~BJ < 0.05; KY~FG < 0.01; LL~FG < 0.05

Notes: The symbol “~” connecting two items indicates that the two are compared pairwise. For example, LL~XF < 0.05 indicates that the *p*-value between the LL and XF groups is <0.05. The rest can be interpreted accordingly.

## Data Availability

The original contributions presented in this study are included in the article/supplementary material. Further inquiries can be directed to the corresponding authors.

## Supplementary Materials

The following supporting information can be downloaded at: https://www.mdpi.com/article/10.3390/foods15111943/s1, Table S1: The detection limits for each mineral in honey; Table S2: Free amino acid content of Chinese gallnut honey from 10 regions in Guizhou Province (μg/g, mean ± SD) (n = 3); Table S3: Proportion of amino acids in honey samples (%); Table S4: Vitamin content of Chinese gallnut honey from 10 regions in Guizhou Province (μg/g, mean ± SD) (n = 3); Table S5: Mineral content of Chinese gallnut honey from 10 regions in Guizhou Province (μg/g, mean ± SD) (n = 3).
