# Supplementary material for: Analysis of Amino Acid, Vitamin, and Mineral Content in Chinese Gallnut (Rhus chinensis Mill.) Honey from Guizhou Province
Source: Foods. 2026 Jun 1;15(11):1943. doi: 10.3390/foods15111943 (PMC13257414; doi:10.3390/foods15111943)
Supplement: Supplementary file 1 [file foods-15-01943-s001.zip › foods-4304741-supplementary.pdf]

Supplementary Materials

Supplementary Table S1. The detection limits for each mineral in honey.

Items	Standard No.	Items	Standard No.
Free amino acids	SN/T 5223—2019	Vitamin E	GB 5009.82-2016
Calcium	GB 5009.92-2016	Vitamin B_6	GB 5009.154-2016
Iron	GB 5009.90-2016	Vitamin D_3	GB 5009.82-2016
Magnesium	GB 5009.241-2017	Vitamin B_1	GB 5009.84-2016
Zinc	GB 5009.14-2017	Vitamin B_2	GB 5009.85-2016
Copper	GB 5009.13-2017	Vitamin B_{12}	GB 5009.285-2022
Potassium, Sodium	GB 5009.91-2017	Niacin	GB 5009.89-2016
Manganese	GB 5009.242-2017	Pantothenic acid	GB 5009.210-2016
Phosphorus	GB 5009.87-2016	Biotin	GB 5009.259-2016
Selenium	GB 5009.93-2017	Folic acid / Folate	GB 5009.211-2022
α-Tocopherol	GB 5009.82-2016	β-Carotene	GB 5009.83-2016
γ-Tocopherol	GB 5009.82-2016	Vitamin K_1	GB 5009.158-2016
δ-Tocopherol	GB 5009.82-2016	Total L(+)-ascorbic acid	GB 5009.86-2016
Vitamin A	GB 5009.82-2016		

Supplementary Table S2. Free amino acid content of Chinese gallnut honey from 10 regions in Guizhou Province (μg/g, mean ± SD) (n=3).

Sites	BJ	DY	DS	FG	KY	LL	MJ	TJ	XF	ZA
Phe	1680.39±14.93	958.38±25.92	589.75±11.99	62.07±2.07	809.57±19.25	1385.91±19.8	818.46±10.71	962.85±6.45	64.91±0.63	1403.76±1.26
Pro	696.19±5.45	136.19±3.67	113.97±3.60	239.14±7.57	180.65±3.30	154.91±2.98	177.09±2.66	235.32±1.06	146.55±2.05	434.06±0.92
Met	212.32±8.42	129.57±9.34	54.35±1.04	314.12±7.54	16.14±3.04	61.24±2.02	105.78±1.93	168.16±8.92	111.91±3.26	382.95±21.22
Ile	134.34±0.97	55.69±1.46	40.23±0.92	141.30±4.76	12.89±1.10	38.3±0.52	73.95±1.24	66.73±0.37	56.03±0.66	123.70±0.59
Asn	111.69±3.01	32.12±0.75	46.32±0.78	89.86±2.97	4.51±0.59	3.16±0.18	37.98±1.51	40.74±1.24	51.55±1.19	98.05±8.15
Tyr	19.86±1.28	12.08±3.18	312.49±6.56	3.15±0.11	4.83±0.98	26.57±1.56	4.10±0.73	43.56±1.52	2.11±0.33	32.82±0.37
Glu	40.79±1.40	11.55±0.47	35.98±1.78	28.53±1.98	11.62±0.36	15.69±0.19	22.22±0.28	23.16±0.63	9.60±1.50	82.70±4.23
Asp	7.69±2.78	3.30±0.41	72.50±7.46	9.01±4.82	9.24±0.65	135.23±6.58	21.25±2.42	8.31±1.16	7.90±0.48	16.51±5.86
Lys	42.26±0.31	17.62±0.29	20.50±0.46	19.54±1.04	11.78±0.55	18.72±0.59	18.77±0.42	22.14±0.43	19.36±0.55	27.97±0.12
Ser	19.33±0.73	2.73±0.37	17.19±1.05	7.56±1.39	59.94±1.39	8.92±0.87	16.61±0.30	8.48±0.64	6.16±0.63	56.09±0.20
Gly	23.75±0.45	4.22±0.99	2.92±0.56	9.28±1.23	30.32±2.53	2.01±0.83	6.46±0.11	6.84±0.51	5.82±0.77	26.05±0.12
Leu	8.27±0.05	6.29±0.10	13.60±0.43	3.41±0.44	7.38±0.36	34.36±0.63	6.16±0.28	13.56±0.11	2.63±0.05	10.68±0.29
Cys	3.31±0.21	14.94±2.1	2.46±0.18	40.54±1.21	4.19±0.22	2.37±0.45	21.90±0.23	1.86±0.85	1.12±0.17	2.64±0.15
His	28.07±2.01	3.96±0.53	2.50±1.76	7.53±0.39	4.36±0.42	1.03±0.89	4.60±0.29	4.76±0.12	2.47±0.18	12.53±1.39
Arg	4.51±2.39	12.29±4.22	8.13±2.23	8.32±5.73	3.49±0.53	12.45±3.93	3.78±0.57	5.44±2.88	4.28±0.87	3.65±0.38
Gln	6.91±0.51	2.81±0.32	14.50±2.15	4.01±0.30	3.01±0.17	0.45±0.57	3.00±0.20	9.98±0.08	1.16±0.05	12.78±0.15
Val	9.95±0.43	6.69±0.43	4.25±0.19	4.33±1.81	3.43±0.28	3.69±0.21	2.64±0.37	6.39±0.19	7.06±0.25	8.49±0.30
Thr	3.54±2.06	1.53±0.64	7.85±0.37	5.07±1.37	5.50±0.69	1.64±0.10	1.42±0.31	3.68±0.69	3.93±0.39	16.63±2.92
Ala	3.77±5.20	5.31±0.80	3.39±3.05	5.09±0.55	6.38±0.72	3.07±0.74	1.41±0.23	5.88±0.14	2.47±0.61	7.49±8.07
Total	3056.94±32.88	1417.26±51.15	1362.89±33.52	1001.85±12.12	1189.23±31.92	1909.72±26.18	1347.59±16.23	1637.84±14.79	507.01±3.98	2759.54±35.48

Notes: Longli County (LL), Dushan County (DS), Taijiang County (TJ), Duyun City (DY), Xifeng County (XF), Fenggang County (FG), Bijiang District (BJ), Majiang County (MJ), Zheng'an County (ZA), and Kaiyang County (KY).

Supplementary Table S3．Proportion of amino acids in honey samples (%).

Amino acid	BJ	DY	DS	FG	KY	LL	MJ	TJ	XF	ZA
Pro	22.77	9.61	8.36	23.87	15.19	8.11	13.14	14.37	28.90	15.73
Phe	54.97	67.62	43.27	6.20	68.08	72.57	60.73	58.79	12.80	50.87
Met	6.95	9.14	3.99	31.35	1.36	3.21	7.85	10.27	22.07	13.88
Ile	4.39	3.93	2.95	14.10	1.08	2.01	5.49	4.07	11.05	4.48
Asn	3.65	2.27	3.40	8.97	0.38	0.17	2.82	2.49	10.17	3.55
Tyr	0.65	0.85	22.93	0.31	0.41	1.39	0.30	2.66	0.42	1.19
Glu	1.33	0.81	2.64	2.85	0.98	0.82	1.65	1.41	1.89	3.00
Asp	0.25	0.23	5.32	0.90	0.78	7.08	1.58	0.51	1.56	0.60
Lys	1.38	1.24	1.50	1.95	0.99	0.98	1.39	1.35	3.82	1.01
Ser	0.63	0.19	1.26	0.75	5.04	0.47	1.23	0.52	1.21	2.03
Gly	0.78	0.30	0.21	0.93	2.55	0.11	0.48	0.42	1.15	0.94
Leu	0.27	0.44	1.00	0.34	0.62	1.80	0.46	0.83	0.52	0.39
Cys	0.11	1.05	0.18	4.05	0.35	0.12	1.63	0.11	0.22	0.10
His	0.92	0.28	0.18	0.75	0.37	0.05	0.34	0.29	0.49	0.45
Arg	0.15	0.87	0.60	0.83	0.29	0.65	0.28	0.33	0.84	0.13
Gln	0.23	0.20	1.06	0.40	0.25	0.02	0.22	0.61	0.23	0.46
Val	0.33	0.47	0.31	0.43	0.29	0.19	0.20	0.39	1.39	0.31
Thr	0.12	0.11	0.58	0.51	0.46	0.09	0.11	0.22	0.78	0.60
Ala	0.12	0.37	0.25	0.51	0.54	0.16	0.10	0.36	0.49	0.27

Notes: Longli County (LL), Dushan County (DS), Taijiang County (TJ), Duyun City (DY), Xifeng County (XF), Fenggang County (FG), Bijiang District (BJ), Majiang County (MJ), Zheng'an County (ZA), and Kaiyang County (KY). N.D. indicates not detected.

Supplementary Table S4. Vitamin content of Chinese gallnut honey from 10 regions in Guizhou Province (μg/g, mean ± SD) (n=3).

Type	BJ	DY	DS	FG	KY	LL	MJ	TJ	XF	ZA
Ascorbic acid	143.41 ± 0.87	70.57 ± 1.03	79.95 ± 1.24	90.99 ± 0.58	39.3 ± 0.54	104.66 ± 2.57	108.7 ± 0.71	138.93 ± 3.25	6.22 ± 0.42	120.03 ± 0.12
Niacin	0.64 ± 0.02	1.75 ± 0.25	1.5 ± 0.14	1.02 ± 0.02	1.18 ± 0.12	2.35 ± 0.17	2.66 ± 0.09	2.59 ± 0.07	1.72 ± 0.12	0.65 ± 0.07
Folic acid	3.13 ± 0.09	6.05 ± 0.1	56.05 ± 1.1	0.36 ± 0.03	6.7 ± 0.32	4.94 ± 0.07	26.64 ± 0.55	6.67 ± 0.29	1.6 ± 0.13	27.37 ± 2.2
Vitamin B_6	28.91 ± 0.48	5.61 ± 0.29	1.95 ± 0.05	130.49 ± 0.81	3.76 ± 0.09	4.42 ± 0.28	25.63 ± 0.32	11.53 ± 0.34	7.89 ± 0.12	36.04 ± 1
Gamma-tocopherol	0.85 ± 0.02	0.26 ± 0.01	0.12 ± 0.01	0.11 ± 0.01	0.31 ± 0.01	0.25 ± 0	0.24 ± 0.01	1.42 ± 0.04	0.12 ± 0.01	0.23 ± 0.02
Vitamin E	1.93 ± 0.03	0.69 ± 0.01	0.29 ± 0.02	0.11 ± 0.01	0.86 ± 0.02	0.81 ± 0.02	0.64 ± 0.01	3 ± 0.08	0.98 ± 0.02	0.66 ± 0.03
Alpha-tocopherol	0.4 ± 0.01	0.05 ± 0	N.D.	N.D.	0.11 ± 0.01	0.17 ± 0.02	0.07 ± 0.01	0.81 ± 0.04	0.82 ± 0.02	N.D.
Carotene	5.64 ± 0.13	1.77 ± 0.06	0.19 ± 0.01	N.D.	0.43 ± 0.02	0.89 ± 0.02	0.73 ± 0.03	14.71 ± 0.21	0.16 ± 0.01	0.34 ± 0.03
Delta-tocopherol	0.68 ± 0.02	0.37 ± 0.01	0.17 ± 0.01	N.D.	0.43 ± 0.01	0.39 ± 0.01	0.33 ± 0.02	0.77 ± 0.02	0.03 ± 0.01	0.43 ± 0.01
Vitamin H	30.78 ± 0.69	34.59 ± 0.94	N.D.	197.18 ± 1.85	4.55 ± 0.1	26.26 ± 0.63	17.12 ± 0.54	28.38 ± 1.37	1.47 ± 0.14	211.14 ± 0.85
Vitamin A	N.D.	2.84 ± 0.08	1.98 ± 0.23	N.D.	0.89 ± 0.17	1.51 ± 1.31	2.07 ± 0.21	2.93 ± 0.55	N.D.	N.D.
Pantothenic acid	3.87 ± 0.07	N.D.	0.15 ± 0.02	3.15 ± 0.25	2.06 ± 0.06	0.22 ± 0.02	2.17 ± 0.07	N.D.	N.D.	4.38 ± 0.05
Vitamin B_{12}	N.D.	0.08 ± 0.02	N.D.	0.26 ± 0.01	N.D.	N.D.	N.D.	0.27 ± 0.02	N.D.	N.D.
Vitamin B_2	4.21 ± 0.2	0.72 ± 0.02	N.D.	5.73 ± 0.23	N.D.	N.D.	10.57 ± 0.5	0.83 ± 0.03	0.91 ± 0.02	2.41 ± 0.28
Vitamin K	N.D.	N.D.	N.D.	N.D.	N.D.	N.D.	N.D.	N.D.	N.D.	N.D.
Vitamin B_1	N.D.	N.D.	N.D.	N.D.	N.D.	N.D.	N.D.	N.D.	N.D.	N.D.
Vitamin D_3	N.D.	N.D.	N.D.	N.D.	N.D.	N.D.	N.D.	N.D.	N.D.	N.D.

Notes: Longli County (LL), Dushan County (DS), Taijiang County (TJ), Duyun City (DY), Xifeng County (XF), Fenggang County (FG), Bijiang District (BJ), Majiang County (MJ), Zheng'an County (ZA), and Kaiyang County (KY). N.D. stands for below the detection limit.

Supplementary Table S5. Mineral content of Chinese gallnut honey from 10 regions in Guizhou Province (μg/g, mean ± SD) (n=3).

Sites	BJ	DY	DS	FG	KY	LL	MJ	TJ	XF	ZA
Mg	27.82±0.54	15.67±0.22	15.75±0.15	27.84±0.30	9.99±0.20	9.84±0.12	18.66±0.47	32.88±0.31	35.95±0.22	35.28±0.34
Mn	0.17±0.01	0.68±0.03	0.51±0.02	1.90±0.02	0.35±0.02	0.30±0.01	1.83±0.07	0.76±0.03	0.92±0.01	0.80±0.03
Fe	4.87±0.12	4.59±0.11	4.03±0.07	4.48±0.05	3.53±0.10	5.64±0.01	4.98±0.14	6.81±0.03	4.87±0.12	4.92±0.21
Cu	0.13±0.01	0.05±0.01	0.07±0	0.07±0.01	0.1±0	0.07±0	0.10±0	0.16±0.01	0.08±0.01	0.13±0.01
Zn	2.05±0.06	1.34±0.02	1.57±0.05	1.60±0.07	1.19±0.02	1.49±0.04	1.33±0.04	1.98±0.07	1.87±0.04	1.94±0.09
Na	19.41±1.40	12.95±0.02	12.43±0.02	20.32±1.92	19.71±1.53	12.84±0.08	20.98±0.1	12.87±0.09	17.79±1.69	18.75±0.08
K	540.33±7.20	466.23±6.08	428.16±0.69	328.79±4.54	469.77±8.3	397.45±6.60	430.29±3.04	489.74±5.38	141.17±3.99	470.06±7.24
Ca	93.89±1.40	61.81±1.78	44.39±0.13	103.28±0.46	61.99±0.86	58.48±2.17	48.02±0.67	77.23±3.29	64.33±0.61	55.61±0.17
P	194.14±5.72	59.88±0.71	91.45±2.17	231.98±9.05	54.20±1.14	58.92±0.54	107.63±1.09	124.98±2.40	114.95±1.96	83.92±1.42
Total	98.09±1.51	69.25±0.58	66.48±0.29	80.03±1.27	68.98±0.71	60.56±0.74	70.42±0.37	83.05±1.09	42.44±0.37	74.60±0.85

Notes: Longli County (LL), Dushan County (DS), Taijiang County (TJ), Duyun City (DY), Xifeng County (XF), Fenggang County (FG), Bijiang District (BJ), Majiang County (MJ), Zheng'an County (ZA), and Kaiyang County (KY).
